# Chloroplast Genome Features and Phylogeny of Two Nationally Protected Medicinal Plants, *Euchresta tubulosa* and *Euchresta japonica*: Molecular Resources for Identification and Conservation

**DOI:** 10.3390/genes16111286

**Published:** 2025-10-29

**Authors:** Dabao Yin, Xue Li, Zhongchun Xiao, Li Zhou

**Affiliations:** Key Laboratory of Biogenetic Resources Mining and Molecular Breeding in Qianxinan Prefecture, Minzu College of Biology and Chemistry, Normal University of Xingyi, Xingyi 562400, China; yindabao717@163.com (D.Y.); 13984663513@163.com (X.L.); xzc729609100@163.com (Z.X.)

**Keywords:** *Euchresta tubulosa*, *Euchresta japonica*, chloroplast genome, SSR, hypervariable region, DNA barcode, phylogeny, medicinal plant conservation

## Abstract

[Objectives]: By performing genome assembly, annotation, comparative characterization, and phylogenetic analysis on the complete chloroplast genomes of *E. tubulosa* and *E. japonica*—two medicinal plants belonging to the genus *Euchresta*—this study aims to identify their differential genes, thereby providing fundamental research for screening candidate genes as DNA barcodes for species identification and facilitating the conservation of these endangered species. [Methods]: Illumina PE150 sequencing was performed. Chloroplast genomes (plastomes) were assembled and annotated with GetOrganelle/SPAdes. Comparative analyses assessed gene content, IR/LSC/SSC structure, repeat profiles, and codon-usage bias. Using related *Fabaceae* as references, we conducted mVISTA alignments and sliding-window nucleotide diversity (Pi) analyses to identify candidate DNA barcodes. Phylogenies from whole-plastome sequences were inferred with Maximum Likelihood, Bayesian Inference, and Maximum Parsimony. [Results]: The plastomes measured 153,960 bp (*E. japonica*) and 150,146 bp (*E. tubulosa*), with GC contents of 36.30% and 36.20%, respectively, each exhibiting a typical quadripartite structure. IR/SC boundaries were highly conserved without evident expansion or contraction. Repeat statistics were 20/30 palindromic repeats, 57/64 tandem repeats, and 156/159 simple sequence repeats (SSRs) in *E. japonica/E. tubulosa*, respectively. Leucine was the most frequently encoded amino acid, cysteine the least, and codon usage favored A/U at third positions. Five hypervariable loci—*rps19*, *psbA*, *trnK*, *matK*, and *rps16* (Pi > 0.03)—were identified as candidate DNA barcodes. All trees consistently placed both species within Papilionoideae (Fabaceae) and recovered the closest relationship to Sophora macrocarpa. [Conclusions]: This study provides, for the first time, complete plastomes and candidate barcoding regions for two protected *Euchresta* species, supplying foundational resources for species identification, resource assessment, and conservation planning.

## 1. Introduction

The genus *Euchresta* comprises four species and three varieties worldwide, with four species and two varieties reported from China. Among them, *Euchresta japonica* Hook. f. ex Regel and *Euchresta tubulosa* Dunn are valued medicinal plants in Tujia ethnomedicine [1]. Modern pharmacological studies have identified alkaloids, flavonoids, and triterpenoid saponins as major constituents of *E. japonica*. Notably, flavonoids including vitexin, calycosin, and formononetin exhibit anti-inflammatory and antitumor activities [2], whereas alkaloids such as cytisine, N-methylcytisine, matrine, and oxymatrine display diverse pharmacological effects, including antitumor, anti-inflammatory, antioxidant, and immunomodulatory properties [3,4,5]. The closely related *E. tubulosa* is likewise used in folk practice for similar indications and is considered to have comparable medicinal value [6]. In traditional applications, both *Euchresta* species are frequently used in the management of cancers such as laryngeal, esophageal, and nasopharyngeal cancer [7,8].

Driven by high medicinal demand, over-harvesting has led to the depletion of wild populations, rendering both species rare and endangered. They have been included in the National Key Protected Wild Plants List of China and classified as threatened species on the Red List of the International Union for Conservation of Nature (IUCN) [9,10]. Beyond the challenges of species conservation, *E. japonica* (Japanese Shandougen) and *E. tubulosa* (Guane Shandougen) are often misused with the authentic Shandougen medicinal material (the dried roots of *Sophora tonkinensis*) in some regions, as they are commonly known by the names “Shandougen” and “Hudoulian” [11]. The confusion of “homonym but heterospecific” is particularly prominent in the Shandougen category of Chinese medicinal materials. According to literature records, there are no fewer than 20 species of medicinal materials circulating under the name “Shandougen” across different regions. This issue of mixed origins has long plagued the Chinese medicinal material market and clinical applications [12]. The overlapping therapeutic effects of these two species with *Sophora tonkinensis* have further exacerbated the adulteration and misuse of medicinal materials [13]. Therefore, it is of practical significance to develop an effective molecular identification technique for the authentication of these two medicinal materials [14].

*E. japonica* is distributed mainly south of the Yangtze River Basin—including Hunan, Hubei, Zhejiang, Jiangxi, Fujian, Guangdong, Guangxi, Guizhou, Sichuan, and Yunnan—typically inhabiting humid riparian valleys and understory environments [8]. By contrast, *E. tubulosa* has a more restricted range, occurring primarily in Hunan, Hubei, Sichuan, Guizhou, and Yunnan [15]. In recent years, habitat degradation has reduced suitable habitats for both species. Together with a low natural reproductive rate in *E. japonica* and persistent harvesting pressure, wild populations have declined sharply, bringing the species to the brink of extinction [16]. Ongoing habitat fragmentation and population contraction highlight the urgency of implementing evidence-based conservation strategies.

Chloroplasts are specialized energy-converting organelles unique to higher plants and certain algae, playing a pivotal role by harboring autonomous genetic information essential for cellular functions [17]. The chloroplast genome has emerged as a powerful tool in plant phylogenetic research, species identification, and DNA barcode development, attributed to its conserved structure, small molecular size, uniparental inheritance, and moderate evolutionary rate [18,19]. In angiosperms, the maternal inheritance of the chloroplast genome contributes to maintaining the stability of species evolution [20], while the occurrence of mutations provides invaluable insights for evolutionary studies [21] and population classification [22]. These mutations also serve as effective genetic markers for unraveling complex evolutionary processes, making chloroplast genes ideal subjects for investigating species evolution [23]. Studies on chloroplast genome barcoding provide an effective technical method for species identification. The *trnQ-rps16* marker enables species-level classification of 7 tested Panax species and 1 unidentified species [24]. The *trnH-psbA* marker is an effective locus for distinguishing Colchicum species [25].

Despite rapid advances in plastome technologies, coverage within the diverse family Fabaceae remains uneven. Public databases are dominated by economically important legumes such as soybean and common bean, whereas genomic resources for rare and endangered genera, including *Euchresta*, remain severely limited [26]. Researchers have successively developed microsatellite markers for *E. japonica* (a species of the *Euchresta* genus) [27,28], and subsequent studies have analyzed the chloroplast genome characteristics of *E. tubulosa* and conducted phylogenetic analyses [29]. However, these studies are insufficient to meet the needs for species identification and conservation of *Euchresta* plants.

Here, we use Illumina PE150 sequencing to characterize the plastomes of the rare and endangered *Euchresta* species *E. japonica* and *E. tubulosa*. Our aims were threefold: (i) to analyze plastome features and structural evolution in both species—providing the complete sequencing, assembly, and annotation of their plastomes—and, through detailed comparisons of genome structure, gene content, repetitive elements, and inverted repeat (IR) boundary dynamics, to elucidate their molecular evolutionary characteristics; (ii) to identify hypervariable regions suitable as species-specific DNA barcodes, thereby enabling accurate discrimination of *E. japonica*, *E. tubulosa*, and common adulterants and supporting market supervision and authentication; and (iii) to clarify the phylogenetic placement of *Euchresta* and provide a genomic basis for delimiting species boundaries and prioritizing conservation actions based on genetic divergence.

## 2. Materials and Methods

### 2.1. Material Collection and DNA Extraction

**Figure 1:**
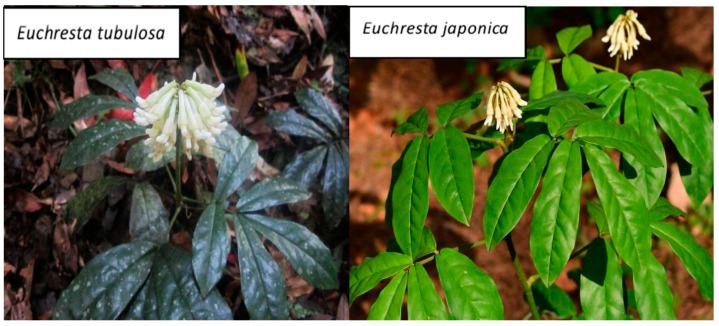
Photos of *Euchresta tubulosa* and *Euchresta japonica*.

In May 2025, healthy young leaf specimens of two individual plants belonging to the genus *Euchresta* (Fabaceae) were collected from Fanjing Mountain, Guizhou Province, China (108°42′ E, 18°53′ N). The collected leaves of the two *Euchresta* species were first carefully rinsed with distilled water, followed by blotting the surface moisture dry with absorbent paper. The treated leaves were then separately placed into cryovials, which were immediately subjected to freezing treatment in liquid nitrogen. After freezing, the specimens were stored in an ultra-low temperature refrigerator at −80 °C for subsequent experimental analysis. The morphological characteristics of these wild specimens are illustrated in Figure 1. The species identification of the plant specimens was conducted by Lina Zhang, and the voucher specimens are currently deposited in the Herbarium of Hainan University. The specimen serial numbers of *E. japonica* and *E. tubulosa* are ZL-202505-GZFJ-01 and ZL-202505-GZFJ-02, respectively.

High-quality genomic DNA was extracted from the leaves using a modified cetyltrimethylammonium bromide (CTAB) method [30]. The concentration and quality of the total DNA were detected by 1% agarose gel electrophoresis. After passing the quality inspection, paired-end 150 bp (PE150) sequencing was performed on the Illumina NovaSeq 6000 platform, with a target raw data volume of ≥3–5 Gb per sample.

The sequencing data were purified using fastp v.0.23.2 software: bases with an average quality score lower than Q20 (error rate ≤ 1%) were removed, and reads with an N-base proportion exceeding 5% were discarded. During the quality control process, key indicators of Clean Data were statistically analyzed, including the percentages of Q20 (base recognition accuracy ≥ 99%) and Q30 (base recognition accuracy ≥ 99.9%), GC content, and read length distribution, to comprehensively evaluate data quality.

### 2.2. Chloroplast Genome Assembly and Annotation

The raw sequencing data generated by Illumina HiSeq were assembled using GetOrganelle software v1.7.7 with specific parameters (−k = 21, 45, 65, 85,105, 121; −t = 128; −R = 15; −F = embplant_pt) to construct the circular plastid genome. The initial assembly results were self-corrected using the Hammer algorithm in SPAdes v3.14.1 [31] under the “careful” mode with default k-mers. Subsequently, Bandage software v3.15.5 [32] was used to verify the circularity of the assembly results, and the coverage depth map was generated following the official protocol.

The assembled chloroplast genomes were annotated using the online platforms CPGAVAS2 and GeSeq (https://chlorobox.mpimp-golm.mpg.de/OGDraw.html, accessed on 1 June 2025) [33]. tRNA genes were detected with tRNAscan-SE [34], and Geneious Prime software 2024.2.1 [35] was employed for in-depth analysis of the chloroplast genome characteristics of the samples, including genome length, lengths and GC contents of the four regions, gene types, and gene copy numbers. The chloroplast genome map was drawn using the online OGDRAW software v2.0.

### 2.3. Repetitive Sequences and SSRs

The online software REPuter [36] (https://bibiserv.cebitec.uni-bielefeld.de/reputer, accessed on 1 June 2025) was used to detect dispersed repetitive sequences in the chloroplast genome, including forward repeats (F), reverse repeats (R), complementary repeats (C), and palindromic repeats (P). The parameters for identifying dispersed repetitive sequences in the genome were set as follows: Hamming distance = 3, minimum repeat length ≥ 11 bp, and other parameters set to default. Only repetitive sequences with an e-value ≤ 1 × 10^−5^ were retained.

Tandem Repeats Finder [37] was used to efficiently detect cryptic repeat units in the DNA sequences, thereby analyzing genome structural variations and evolutionary dynamics. MISA software [38] was applied for simple sequence repeat (SSR) analysis, with the minimum repeat parameters set as follows: mononucleotides > 10, dinucleotides > 5, trinucleotides ≥ 4, and tetra-/penta-/hexanucleotides ≥ 3.

### 2.4. Codon Preference

Codon usage bias (CUB) analysis was performed on non-redundant protein-coding genes (PCGs) [39]. The relative synonymous codon usage (RSCU) values were calculated using CodonW software 1.4.4 [40]. Heatmaps or radar charts of the RSCU values were generated using interactive visualization platforms such as ChiPlot or custom scripts (e.g., ggplot2 based on R language).

### 2.5. Structural Alignment and Variation Hotspots

Thirteen publicly available chloroplast genomes of congeneric related species were retrieved from the NCBI database. The genome files were converted into a format compatible with mVISTA (https://genome.lbl.gov/vista/mvista/submit.shtml (accessed on 1 June 2025)). Based on the results of phylogenetic analysis, IRSCOPE (https://irscope.shinyapps.io/irapp/ (accessed on 1 June 2025)) [41] was used to conduct whole-genome alignment analysis of Sophora tonkinensis, Sophora tubicalyx, and the 13 publicly available chloroplast genomes of related species (from NCBI).

DnaSP v6.12.03 software [42] was used to calculate the number of single nucleotide polymorphisms (SNPs) and insertion/deletion variations (InDels), and the mutation frequency within each 100 base pair (bp) interval was counted. Furthermore, the nucleotide diversity (Pi) of the chloroplast genomes of the two *Sophora* species was estimated. A Pi value exceeding 0.03 was defined as a highly variable region.

### 2.6. Phylogenetic Analysis

Chloroplast genome sequences of 19 species from the subfamily Papilionoideae (Fabaceae) and 2 outgroup species (*Astragalus melilotoides* and *Astragalus mongholicus*) were downloaded from the NCBI database. Multiple sequence alignment of the chloroplast genome sequences was performed using MAFFT software [43] within the Phylosuite platform. The alignment results were trimmed using trimAl software [44].

Maximum likelihood (ML) analysis was conducted using RAxML v8.2.10 software, with 1000 replicates of rapid bootstrap testing to assess node support values and construct the phylogenetic tree [45,46]. Two outgroup species, *A. melilotoides* and *A. mongholicus*, were designated to stabilize the root of the phylogenetic tree.

## 3. Results

### 3.1. Composition and Features of the Chloroplast Genome

**Figure 2:**
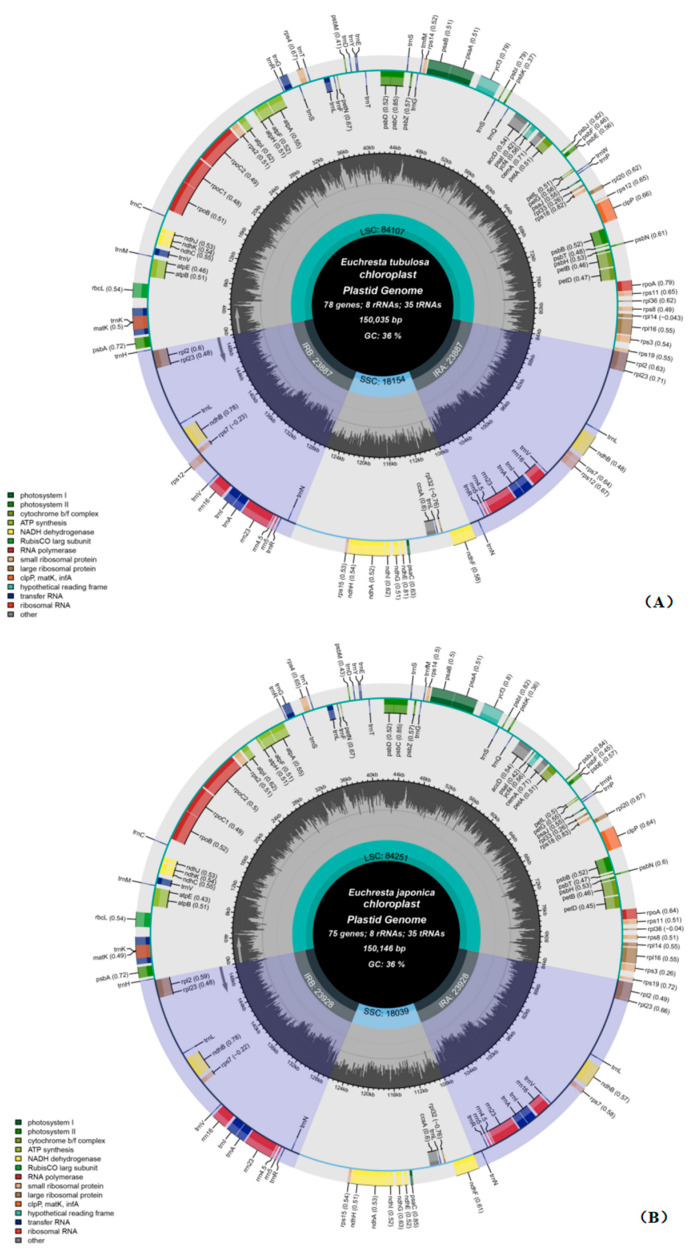
Annotated plastid genome maps of *E. tubulosa* (**A**) and *E. japonica* (**B**). The darker gray in the inner circle represents GC content. Inverted Repeat A (IRA), Inverted Repeat B (IRB), Large Single Copy (LSC) region, and Small Single Copy (SSC) region are labeled outside the GC content circle.

Both *E. tubulosa* and *E. japonica* have typical quadripartite chloroplast genomes, which consist of a large single-copy (LSC) region, a small single-copy (SSC) region, and two inverted repeats (IRs) (Figure 1). The length of the chloroplast genome of *E. tubulosa* is 153,960 bp, and its overall GC content is 36.3% (Figure 2A). The LSC region is 84,107 bp long (with a GC content of 42.63%), the SSC region is 18,053 bp long (33.77% GC), and each IR region is 51,800 bp long (29.80% GC). The chloroplast genome of *E. japonica* is 150,146 bp in length, with an overall GC content of 36.20% (Figure 2B). The LSC region measures 84,251 bp (33.70% GC), the SSC region is 18,039 bp (29.80% GC), and each IR region is 47,856 bp (42.90% GC).

In *E. tubulosa*, the plastome contains 127 unique genes, including 84 protein-coding genes (CDS), 38 tRNA genes, and 8 rRNA genes. Functionally, 72 genes are related to self-replication, 44 are involved in photosynthesis, and 5 have other functions (including *matK, clpP, cemA, accD*, and *ccsA*), and 6 are conserved open reading frames with unknown functions (Table 1). Among the genes related to photosynthesis, *ndhB* has two copies and contains one intron, while *petB* (cytochrome b/f complex) and *atpF* (ATP synthase subunit) each have a single intron. In the self-replication category, the duplicated genes include large-subunit ribosomal proteins (*rpl2*, *rpl23*), small-subunit ribosomal proteins (*rps12*, *rps7*), all four rRNA genes (*rrn16S, rrn23S, rrn4.5S, rrn5S*), and several tRNAs (*trnA-UGC, trnI-CAU, trnI-GAU, trnL-CAA, trnN-GUU*, *trnR-ACG*, and *trnV-GAC*). Single—intron genes include *rpl16, rpl2, rpoC1, trnA-UGC, trnG-UCC, trnI-GAU*, *trnL-UAA*, and *trnV-UAC*, and *clpP* contains two introns. Pseudogenized copies of *ycf1* and *ycf2* exist in duplicate, and *ycf3* has two introns.

### 3.2. IR Boundaries and Structural Variations

**Figure 3:**
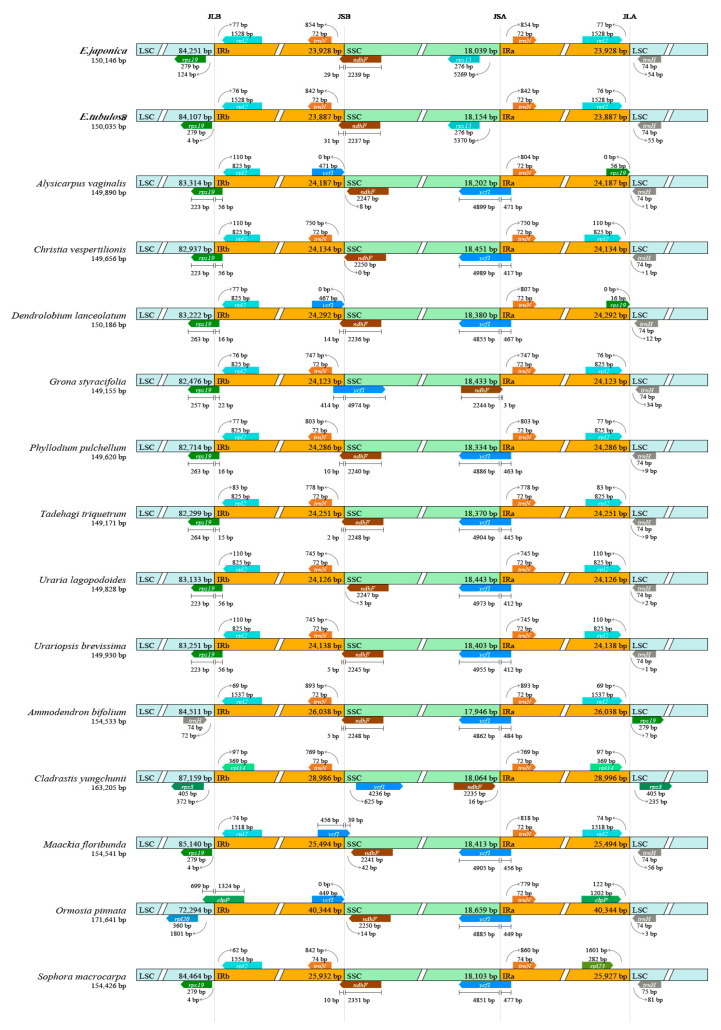
Visual Comparison of Boundaries Between the Large Single-Copy (LSC), Small Single-Copy (SSC), and Inverted Repeat (IR) Regions in the Complete Chloroplast Genomes of 15 Species from the Papilionoideae Subfamily (*Fabaceae*). Junctions between the LSC/SSC regions and inverted repeat regions (IRa, IRb) are defined and labeled as follows: the junction of LSC and IRa is denoted as JLA; the junction of LSC and IRb as JLB; the junction of SSC and IRa as JSA; and the junction of SSC and IRb as JSB. Colored boxes correspond to genes, with the numbers above or below each box representing the base pair distance between the terminal end of the respective gene and the nearest boundary.

The boundaries of the inverted repeat (IR) regions are regarded as hotspots for gene duplication or deletion events, which are crucial in driving variations in chloroplast genome size. In this study, we explored the expansion and contraction dynamics of IR regions in the chloroplast genomes of *E. tubulosa*, *E. japonica*, and 13 other leguminous species from the subfamily Papilionoideae. Our analyses showed that these chloroplast genomes have a high level of conservation in both sequence similarity and overall structural organization (Figure 3). Variations in chloroplast genome size among these species are mainly due to differences in the lengths of the large single-copy (LSC) region, the small single-copy (SSC) region, and the IR regions.

We further conducted a detailed comparison of the exact positions of IR boundaries and their flanking genes across the chloroplast genomes of the studied species. Notably, the *ndhF* gene is located near the SSC/IRb boundary in multiple taxa. For example, in *Dendrolobium lanceolatum*, it is only 14 bp from the boundary, and in *Tadehagi triquetrum*, it is as close as 2 bp. Additionally, the *rpl2* gene is 110 bp away from the LSC/IRb boundary in *Alysicarpus vaginalis*, while in *Phyllodium pulchellum*, this distance is 77 bp, indicating distinct inter-specific differences in the positional arrangement of this gene relative to the IR boundaries [47,48].

The length of the *ycf1* gene varies significantly among species. It is 4899 bp in *Alysicarpus vaginalis*, 4989 bp in *Christia vespertilionis*, 4974 bp in *Grona styracifolia*, and 4885 bp in Ormosia pinnata. Moreover, the *rps19* gene shows length polymorphism across different genera. It is 223 bp long in both *Uraria lagopodoides* and *Urariopsis brevissima*, but extends to 279 bp in *Sophora macrocarpa*. These findings together emphasize the dynamic nature of IR boundary regions in leguminous chloroplast genomes, providing valuable information for future comparative genomics and evolutionary studies.

### 3.3. Collinearity Analysis of the Complete Chloroplast Genome

**Figure 4:**
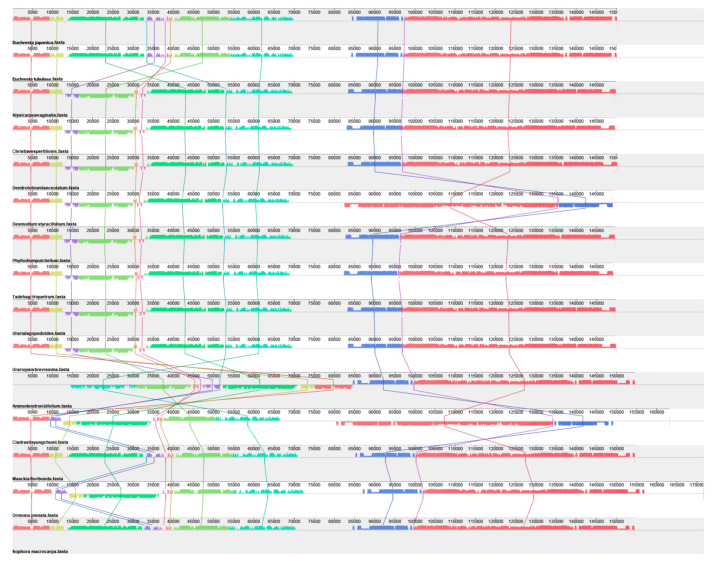
Collinearity Analysis of *E. tubulosa*, *E. japonica*, and 13 Other Species from the Papilionoideae Subfamily (Fabaceae). Regions connected by arcs represent those with significant homology. Red arcs indicate inverted sequences, while gray regions represent forward sequences. Regions without collinear blocks indicate species-specific unique sequences.

Based on chloroplast genome collinearity analysis, this study systematically compared the collinear regions among *E. tubulosa*, *E. japonica*, and 13 other species from the Papilionoideae subfamily (*Fabaceae*) (Figure 4). The results showed that *E. tubulosa* and *E. japonica* shared numerous homologous collinear genome blocks with other *Fabaceae* species, indicating significant genomic conservation of *Euchresta* species within the Papilionoideae subfamily. Among these comparisons, the collinearity between *Euchresta* and *Cladrastis yungchunii* was the most intensive: the total length of collinear blocks reached 292,034 bp, accounting for 58.2% of the total chloroplast genome length of *Euchresta* (assuming its chloroplast genome length is ~500 kb). In contrast, the collinearity between *Euchresta* and *Christia* vespertilionis (genus Christia) was significantly reduced, with collinear blocks accounting for only 12.3% of the *Euchresta* chloroplast genome (total length: 61,500 bp). Notably, almost no collinearity was detected between *Euchresta* and Alysicarpus vaginalis (genus Alysicarpus) (<5%), suggesting that these two taxa may have a distant phylogenetic relationship or have undergone independent genomic evolutionary events.

Regarding genomic structural variations, the arrangement order and orientation of collinear blocks showed significant differences among different species. Compared with Ormosia pinnata (genus Ormosia), 7 large-scale inversion events and 3 translocation events were detected in the *Euchresta* genome. The collinear pattern of *Maackia floribunda* (genus Maackia) differed most significantly from that of *Euchresta*, with approximately 15% of its genomic regions undergoing rearrangement—indicating that this clade may have experienced a unique genomic evolutionary process. Further comparative analysis revealed that, compared with the closely related genus *Sophora*, the intergenic spacer regions (IGS) of *Euchresta* exhibited a length variation rate of 22.5%. Additionally, 8 specific insertions/deletions (Indels) were identified at the boundaries of collinear blocks; these structural variations may affect the regulation or functional differentiation of adjacent genes.

In conclusion, the chloroplast genome of *Euchresta* exhibits high structural plasticity, and its collinearity pattern is characterized by the coexistence of conservation and variation. While maintaining the stability of core genomic regions, *Euchresta* has formed a unique genomic structure through variation mechanisms such as inversion, translocation, and local Indels. This finding provides new insights into understanding the adaptive evolution of Fabaceae plants and lineage-specific genomic remodeling.

### 3.4. Relative Synonymous Codon Usage (RSCU)

The codon usage frequency and relative synonymous codon usage (RSCU) were computed based on the protein-coding genes in the chloroplast genomes of *E*. *tubulosa* and *E*. *japonica*. In this study, a total of 64 codons encoding 20 amino acids were recognized: leucine (Leu), serine (Ser), and arginine (Arg) each had 6 codons; alanine (Ala), proline (Pro), threonine (Thr), valine (Val), and glycine (Gly) each had 4 codons; and isoleucine (Ile) had 3 codons. Among all the protein-coding genes in the chloroplast genome, leucine (Leu) had the highest RSCU value (2.12 ± 0.32%), while phenylalanine (Phe) had the lowest usage frequency (1.41 ± 0.59%) (Figure 4). Generally, codons with RSCU > 1 are more favored. Among the 64 codons, 31 had RSCU > 1, of which only 2 ended with G, and the rest ended with A or U. Methionine (Met) and tryptophan (Trp) were each encoded by a single codon, suggesting that there was no codon usage bias for these two amino acids (RSCU = 1).

### 3.5. Repetitive Sequences and Simple Sequence Repeats (SSRs)

**Figure 5:**
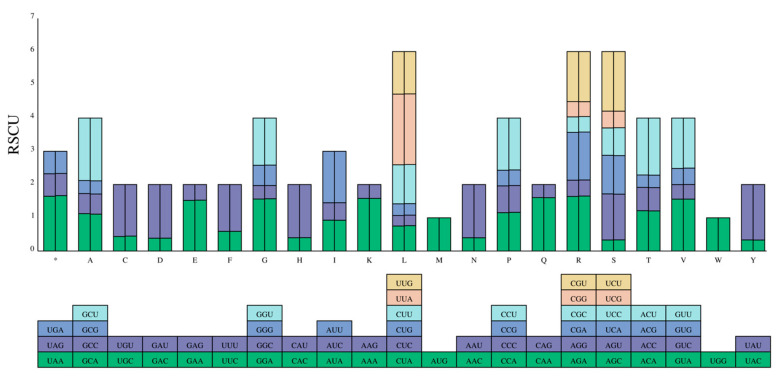
Relative Synonymous Codon Usage (RSCU) in the Chloroplast Genomes of *E. tubulosa* and *E. japonica*. The color of the histograms corresponds to the color of the codons.

**Figure 6:**
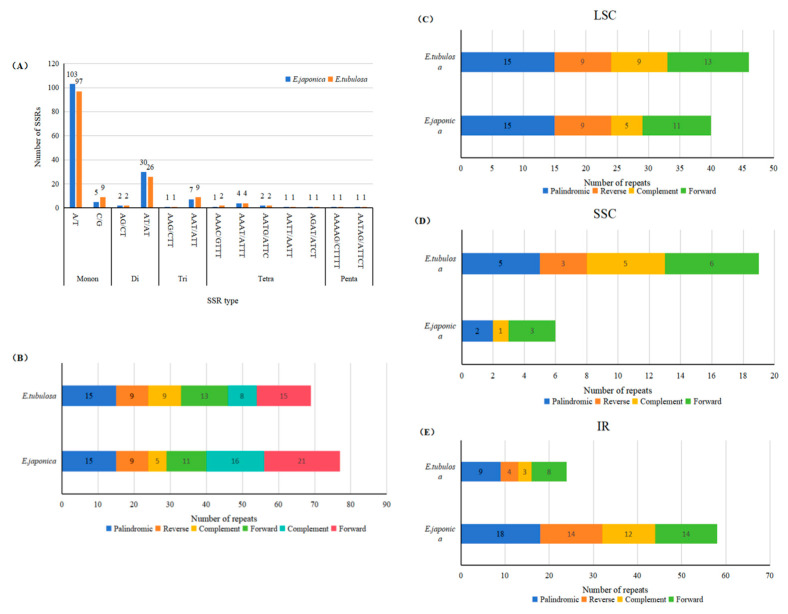
Sequence Repeat Analysis in the Chloroplast Genomes of *E. tubulosa* and *E. japonica*. (**A**). Frequency of different SSR types; (**B**). Frequency of identified long repeat types; (**C**). Frequency of different LSC types; (**D**). Frequency of different SSC types; (**E**). Frequency of different IR types.

A total of 156 simple sequence repeat (SSR) loci were identified in *E. tubulosa*, which included 106 mononucleotide, 28 dinucleotide, 10 trinucleotide, 10 tetranucleotide, and 2 pentanucleotide motifs (Figure 5). For *E. japonica*, 159 SSR loci were detected, composed of 108 mononucleotide, 32 dinucleotide, 8 trinucleotide, 9 tetranucleotide, and 2 pentanucleotide motifs. The most prevalent repetitive motifs were T, A, and AT (Figure 6A).

Regarding the pattern of repetitive motifs in *E. tubulosa*, tandem repeats were dominant (57 loci, approximately 37%), followed by palindromic repeats (20 loci, approximately 13%), forward repeats (15 loci, approximately 10%), inverted repeats (7 loci, approximately 4%), and complement repeats (8 loci, approximately 5%) (Figure 4). The most common repetitive motifs were T, A, and AT. For *E. japonica*, the motif pattern showed that tandem repeats were also dominant (64 loci, approximately 40%), followed by palindromic repeats (30 loci, approximately 19%), forward repeats (21 loci, approximately 13%), inverted repeats (23 loci, approximately 14%), and complement repeats (16 loci, approximately 10%) (Figure 6B).

These findings suggest that SSRs and long repetitive sequences show inter-specific differences, which offer opportunities for developing new molecular markers for the identification of *E. tubulosa* and *E. japonica*. An analysis of the number of low-abundance repeat sequence types in the chloroplast genomes of the two species revealed that in the LSC region, the most significant difference was observed in the Complement type, with *E. tubulosa* (9.0) having a higher count than *E. japonica* (5.0). In the SSC region, the Reverse type showed the most prominent difference between the two species: it was absent in *E. japonica* (0.0) but present in *E. tubulosa* (3.0). For the Palindromic type, *E. tubulosa* (5.0) had a higher count than *E. japonica* (2.0). In the Complement type, *E. tubulosa* (5.0) had a considerably higher count compared to *E. japonica* (1.0). Regarding the Forward type, *E. tubulosa* (6.0) also exceeded *E. japonica* (3.0). This figure focuses on low-abundance repeats, highlighting differences in rare repeat types between the two species. Despite their small amounts, such differences may have species-specific characteristics and could potentially serve as unique molecular markers for the accurate differentiation of *E. japonica* and *E. tubulosa*.

In the comparison of low-abundance repeats in the IR region, all repeat types had higher counts in *E. japonica* than in *E. tubulosa*. For the Palindromic type, the count in *E. japonica* (18.0) was twice that in *E. tubulosa* (9.0). In the Reverse type, *E. japonica* (14.0) had a 3.5-fold higher count compared to *E. tubulosa* (4.0). For the Complement type, *E. japonica* (12.0) had a 4-fold higher count than *E. tubulosa* (3.0). In the Forward type, *E. japonica* (14.0) had a 1.75-fold higher count compared to *E. tubulosa* (8.0). This figure demonstrates significant differences in high-abundance repeats between the two species, with the Complement type showing the greatest disparity. This type could be prioritized for the development of efficient molecular markers, providing technical support for the rapid identification of these two Iris species.

### 3.6. Nucleotide Diversity

**Figure 7:**
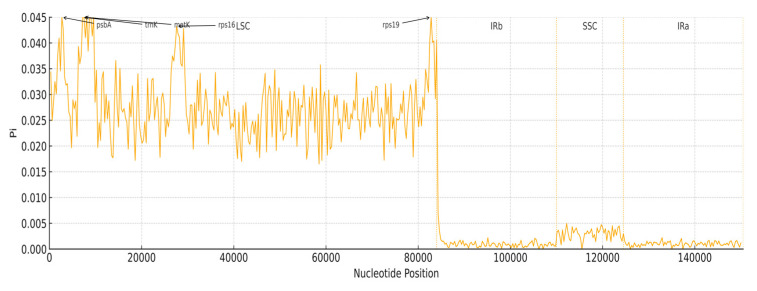
Comparison of Nucleotide Diversity (Pi).

To gain a deeper understanding of the DNA polymorphism (Pi) in the chloroplast genomes of *E. tubulosa* and *E. japonica*, a sliding-window analysis was carried out on the two sets of genomes (Figure 7). The large single-copy (LSC) region had the highest average Pi value of 0.028, while the small single-copy (SSC) region had an average Pi value of 0.003. In contrast, the inverted repeat (IR) regions showed significantly lower nucleotide diversity, with an average Pi value of 0.001. These results indicate that the IR regions are more conserved than the LSC and SSC regions, which is in line with the generally recognized evolutionary pattern of chloroplast genomes. Notably, regions including *rps19, psbA, trnK, matK*, and *rps16* displayed the highest nucleotide diversity. These hypervariable regions can serve as molecular markers for future phylogenetic analysis and species identification of *E. tubulosa* and *E. japonica*.

### 3.7. Phylogenetic Analysis Based on Complete Chloroplast Genomes

**Figure 8:**
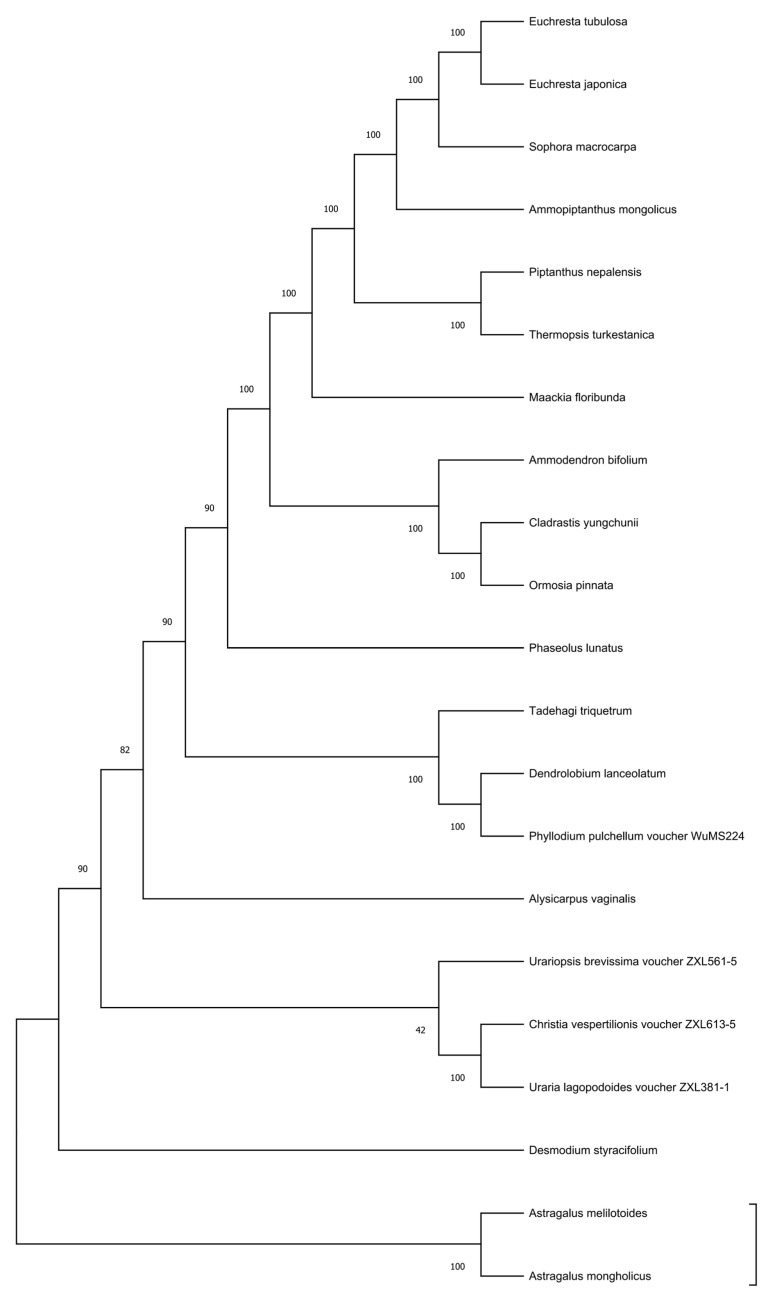
Phylogenetic Tree Based on Chloroplast Genomes.

To clarify the phylogenetic positions of *Euchresta* and its related taxa, phylogenetic analysis was conducted in this study based on 21 complete chloroplast genome sequences, including 19 species from the Papilionoideae subfamily (*Fabaceae*) and 2 outgroup species (Figure 8). After multiple sequence alignment using MAFFT v7, the matrix was optimized and trimmed with trimAl v1.4. Finally, an alignment matrix of 128,756 bp was obtained, containing 15,328 variable sites and 7642 parsimony-informative sites.

Phylogenetic trees constructed using the Maximum Likelihood (ML, IQ-TREE 2.1.2, GTR+F+R8 model, 1000 bootstrap replicates), Maximum Parsimony (MP, PAUP* 4.0, heuristic search), and Bayesian Inference (BI, MrBayes 3.2.7, 2 × 10^6^ generations of Markov Chain Monte Carlo (MCMC)) methods showed a highly consistent topological structure (topological conflict < 3%). The analysis results strongly supported (BSML = 98, BSMP = 95, PP = 1.00) the division of the studied taxa into three major clades: tribe Sophoreae, tribe Dalbergieae, and tribe Phaseoleae.

*E. tubulosa* and *E. japonica* were clearly assigned to tribe *Sophoreae* (BSML = 100, BSMP = 99, PP = 1.00) and formed a stable sister group relationship with *Sophora* macrocarpa. The phylogenetic relationship within tribe *Sophoreae* was resolved as (*Euchresta* (*Sophora* (*Maackia*, *Cladrastis*))), with extremely high statistical support for each node (BSML ≥ 95, BSMP ≥ 93, PP ≥ 0.99). Notably, the clustering relationship of *Ammodendron bifolium* and *Ormosia pinnata* (BSML = 90, BSMP = 88, PP = 0.98) provides new molecular evidence for the inter-tribal relationships in traditional taxonomy.

Figure Legend: Phylogenetic tree of the chloroplast coding regions in Fabaceae. Sequences were aligned using MAFFT v7 (L-INS-i) and trimmed with trimAl (-automated1); partitioning was performed according to genes/codon positions. The partitioned models were selected via ModelFinder in IQ-TREE 2 (with the option of merging partitions using MFP + MERGE), and the phylogenetic tree was inferred based on maximum likelihood. Numbers above the branches represent SH-aLRT support values (1000 replicates), while those below the branches represent UFBoot support values (1000 replicates).

## 4. Discussion

The chloroplast genomes of *E. tubulosa* and *E. japonica* assembled in this study display the canonical quadripartite architecture (LSC + SSC + 2 × IR) [47]. Their lengths (153,960 bp and 150,146 bp) and GC contents (36.20–36.30%) closely match those of related *Papilionoideae taxa*. For example, the plastome of *Sophora macrocarpa* (tribe Sophoreae) is about 152 kb with 36.4% GC, and that of Glycine max is 152,218 bp with 35.9% GC [48]. These comparisons indicate that plastome size and base composition in *Euchresta* fall within the conserved range typical of Fabaceae, reflecting overall structural stability through evolution [49]. In this experiment, single-plant leaves of *E. tubulosa* and *E. japonica* were used as samples. This approach may fail to represent the intraspecific genetic diversity and differences in environmental adaptability of the species, potentially leading to one-sided conclusions.

No marked expansion or contraction of the inverted repeats (IRs) was detected. Boundary-associated genes (e.g., *ndhF, rpl2, ycf1*) show positions comparable to those in *Sophora* and *Maackia*: *ndhF* lies 14–20 bp from the SSC/IRb junction, and *rpl2* is 77–110 bp from the LSC/IRb boundary, consistent with patterns reported across *Papilionoideae* [50,51]. The size (about 3.8 kb) difference between the two *Euchresta* plastomes is therefore attributable mainly to variation within the LSC. Synteny analyses identified eight LSC-specific insertions/deletions, and nucleotide diversity was substantially higher in the LSC (Pi = 0.028) than in the IR (Pi = 0.001), reinforcing the well-established rule of angiosperm plastome evolution-conserved IRs alongside more labile LSC/SSC regions [52].

We detected no large-scale gene rearrangements in either *Euchresta* plastome, in contrast to the extensive structural changes reported for some legume lineages (e.g., Phaseolus) [53]. We hypothesize that the possible reason for this phenomenon is as follows: plants of the genus Euchresta have long adapted to relatively stable and humid understory habitats, and under low-light conditions, the conserved genome architecture may help maintain their photosynthetic efficiency and metabolic integrity [54].

Both species retain a complete complement of the 11 *ndh* genes (*ndhA*–*ndhK*). *ndhB* occurs in duplicate and contains a single intron, and genes such as *ndhA* and *ndhF* show no evidence of pseudogenization. This retention mirrors patterns in many Papilionoideae (e.g., *Sophora*, *Ormosia*) but contrasts with *ndh* loss documented in certain parasitic or hemiparasitic plants (e.g., *Loranthaceae*) [55,56]. The *ndh*-encoded NADH dehydrogenase complex contributes to photosystem I electron transport and cyclic electron flow, processes linked to tolerance of low light, drought, and other stresses [57]. Given that *E. japonica* and *E. tubulosa* occupy shaded, humid microhabitats, we thus hypothesize that the intact set of *ndh* genes may meet their energy demands in understory environments by enhancing cyclic electron flow and ATP synthesis [58]. Future work should test this prediction via expression profiling (e.g., RNA-seq of *ndh* genes) and photosynthetic physiology under controlled light regimes to avoid overinterpretation.

Sliding-window analyses identified five hypervariable loci—*rps19*, *psbA, trnK, matK*, and *rps16*—with Pi > 0.03, exceeding the plastome-wide average (Pi = 0.008) and all located in the LSC [59]. From a marker-selection perspective, *matK* is a core plant DNA barcode widely applied in Fabaceae [60]. Our results further indicate that combining *matK* with *rps19* and *trnK* improves resolution: the *trnK*–*matK* intergenic spacer shows 2.3% divergence between *E. japonica* and *E. tubulosa*, versus 1.5% for *matK* alone, enabling reliable species discrimination. Additionally, *rps16* exhibits 3.1% divergence between *Euchresta* and closely related genera (e.g., *Sophora, Dalbergia*), helping to resolve the long-standing “homonym but heterospecific” issue surrounding “Shandougen.”

Phylogenomic inference using complete plastomes (ML, BI, MP) consistently placed *E. tubulosa* and *E. japonica* within *Sophoreae* and recovered them as sister to *S. macrocarpa*, with strong support (BS_ML = 100; PP = 1.00). The intratribal topology was resolved as (*Euchresta* (*Sophora* (*Maackia*, *Cladrastis*)), thereby clarifying the position of *Euchresta* within *Sophoreae* and challenging earlier morphology-based hypotheses that allied *Euchresta* more closely with *Dalbergia* [61].

## 5. Conclusions

This study focused on the complete chloroplast genomes of *E. tubulosa* and *E. japonica*. It showed high consistency in chloroplast genome size and base composition between these two plants and their close relatives in the Papilionoideae subfamily of Fabaceae. *Sophora tonkinensis* is one such relative. The two species differ in genome size by approximately 3.8 kb. This difference mainly comes from insertions/deletions (indels) of fragments in the Large Single-Copy (LSC) region. Phylogenetic analysis was conducted. It indicated that *E. tubulosa* and *E. japonica* cluster within the Sophoreae tribe of Fabaceae. They also form a stable sister group relationship with *S. tonkinensis*. The phylogenetic relationship within Sophoreae is defined as “*Euchresta* ((*Sophora*, (*Maackia*, *Cladrastis*))”. This revises the early hypothesis. The old hypothesis, based on morphological characteristics, suggested that “*Euchresta* is closely related to *Dalbergia*”. This study reveals the chloroplast genome characteristics of the two *Euchresta* species. It provides key molecular markers and phylogenetic evidence. Additionally, it offers important molecular resources and technical support. These resources support the identification, resource evaluation, and scientific conservation of rare and endangered medicinal plants.

## Figures and Tables

**Table 1:** Gene annotation of the chloroplast genome of *E. tubulosa* and *E*. *japonica.*.

Gene Category	Gene Group	Gene Name
Genes for photosynthesis	Subunits of photosystemI	*psaa*, *psaB*, *psaC*, *psaI*, *psaJ*
	Subunits of photosystemII	*psbA, psbB, psbC, psbD, psbE, psbF, psbH, psbI, psbJ, psbK, psbM, psbN, psbT, psbZ*
	Subunits of NADH dehydrogenase	ndhA *, ndhB *(2), ndhC, ndhD, ndhE, ndhF, ndhG, ndhH, ndhI, ndhJ, ndhK
	Subunit of cytochromeb/f complex	*petA, petB* *, petD *, petG, petL, petN
	Subunits of ATP synthase	*atpA, atpB, atpE, atpF* *, atpH, atpI
	Large subunit of Rubisco	*rbcL*
Self-replication	Proteins of large ribosomal subunit	*rpl14, rpl16* *, rpl2 *(2), rpl20, rpl23(2), rpl32, rpl33, rpl36
	Proteins of small ribosomal subunit	*rps11, rps12* **(2), rps14, rps15, rps16, rps18, rps19, rps2, rps3, rps4, rps7(2), rps8
	Subunits of RNA polymerase	*rpoA, rpoB, rpoC1* *, rpoC2
	Ribosomal RNAs	*rrn16S(2), rrn23S(2), rrn4.5S(2), rrn5S(2)*
	Transfer RNAs	*trnA-UGC* *(2), trnC-GCA, trnD-GUC, trnE-UUC, trnF-GAA, trnG-GCC, trnG-UCC*, trnH-GUG, trnI-CAU(2), trnI-GAU *(2), trnL-CAA(2), trnL-UAA *, trnL-UAG, trnM-CAU, trnN-GUU(2), trnP-GGG, trnQ-UUG, trnR-ACG(2), trnR-UCU, trnS-GCU, trnS-GGA, trnS-UGA, trnT-GGU, trnT-UGU, trnV-GAC(2), trnV-UAC*, trnW-CCA, trnY-GUA, trnfM-CAU
Other genes	Maturase	*matK*
	Protease	*clpP* **
	Envelop membrane proteinn	*cemA*
	acetyl-CoA-carboxylase	*accD*
	c-type cytochrome synthesis gene	*ccsA*
Genes of unknown function	Conserved hypothetical chloroplast ORF	*ycf1(2), ycf2* *(2), ycf3 **, ycf4

## Data Availability

Due to the confidentiality of the project, relevant data cannot be made publicly available at this time. The data will be shared in accordance with the relevant regulations at an appropriate time.
